# Design of an end-effector for robot-assisted ultrasound-guided breast biopsies

**DOI:** 10.1007/s11548-020-02122-1

**Published:** 2020-02-25

**Authors:** Marcel K. Welleweerd, Françoise J. Siepel, Vincent Groenhuis, Jeroen Veltman, Stefano Stramigioli

**Affiliations:** 1 Robotics and Mechatronics, University of Twente, Enschede, The Netherlands; 2 Ziekenhuisgroep Twente, Almelo, The Netherlands; 3 Bio-mechatronics and Energy-Efficient Robotics Group, ITMO University, St. Petersburg, Russian Federation

**Keywords:** End-effector, Robotics, Biopsy, Breast, MRI, Ultrasound, Registration

## Abstract

**Purpose:**

The biopsy procedure is an important phase in breast cancer diagnosis. Accurate breast imaging and precise needle placement are crucial in lesion targeting. This paper presents an end-effector (EE) for robotic 3D ultrasound (US) breast acquisitions and US-guided breast biopsies. The EE mechanically guides the needle to a specified target within the US plane. The needle is controlled in all degrees of freedom (DOFs) except for the direction of insertion, which is controlled by the radiologist. It determines the correct needle depth and stops the needle accordingly.

**Method:**

In the envisioned procedure, a robotic arm performs localization of the breast, 3D US volume acquisition and reconstruction, target identification and needle guidance. Therefore, the EE is equipped with a stereo camera setup, a picobeamer, US probe holder, a three-DOF needle guide and a needle stop. The design was realized by prototyping techniques. Experiments were performed to determine needle placement accuracy in-air. The EE was placed on a seven-DOF robotic manipulator to determine the biopsy accuracy on a cuboid phantom.

**Results:**

Needle placement accuracy was 0.3 ± 1.5 mm in and 0.1 ± 0.36 mm out of the US plane. Needle depth was regulated with an accuracy of 100 µm (maximum error 0.89 mm). The maximum holding force of the stop was approximately 6 N. The system reached a Euclidean distance error of 3.21 mm between the needle tip and the target and a normal distance of 3.03 mm between the needle trajectory and the target.

**Conclusion:**

An all in one solution was presented which, attached to a robotic arm, assists the radiologist in breast cancer imaging and biopsy. It has a high needle placement accuracy, yet the radiologist is in control like in the conventional procedure.

**Electronic supplementary material:**

The online version of this article (10.1007/s11548-020-02122-1) contains supplementary material, which is available to authorized users.

## Introduction

Breast cancer is the most prevalent cancer in women worldwide. In 2018 alone, nearly 2.1 million new cases were diagnosed [1]. It is essential for these women that the diagnosis is confirmed in an early stage of the disease as early detection is known to reduce mortality rates in breast cancer [2].

Several methods are used to detect lesions including self-examination through palpation and imaging modalities such as mammography, ultrasound (US) scans and magnetic resonance imaging (MRI) scans. Mammography is the most common imaging modality in clinical practice. If a lesion is detected, a tissue sample is required to confirm malignancy. This tissue sample is acquired using a biopsy needle, after which the sample is sent to the pathologist. Mostly, the biopsy procedure is performed under US guidance. The radiologist navigates the needle based on US feedback. Disadvantages of this procedure include difficulties in extracting cells from the lesion due to its small size, or poor sensitivity due to difficulties in visualizing tumors against a background of dense fibroglandular tissue [3]. Also, needle insertion is hampered by tissue boundaries and lesion displacement because of forces exerted during needle insertion. The biopsy is repeated if the lesion is not hit at the previous attempt. Consequently, radiologists should be experienced to be successful. However, clinicians who frequently use this technique often suffer from fatigue and work-related musculoskeletal discomfort [4]. These work-related issues will become more frequent since the number of breast biopsies is increasing due to broader access to population screenings for breast cancer.

Robotics can play a major role in these challenges; robots can more accurately, precisely and stably manipulate tools than humans. Moreover, robots do not experience fatigue and consequently the time per patient can be brought down [5]. Furthermore, a robotically steered US probe can produce accurate 3D US volume reconstruction. The US probe position is acquired with high precision utilizing the sensors in the robot, and uniformly spaced slices can be produced with coordinated movements. The accuracy of a biopsy benefits of image fusion of preoperative images is acquired by, e.g., MRI with intra-operative data like US [6]. If the robot “knows” its relative position to the breast and is able to generate a precise 3D US volume, this can ease registration. Because of these advantages, the number of false negatives during a robot-assisted US-guided biopsy is potentially reduced compared to the regular procedure and patient discomfort and cost can be brought down.

Thus, robotic assistance during US-guided breast biopsies is beneficial by providing a stable hand and real-time image feedback. The previous studies focused mainly on the design of mechanisms to assist the radiologist to more accurately perform minimally invasive procedures. Determining the position of the target relative to the biopsy device is an important step in a robot-assisted biopsy. This can be performed by registering preoperative images with the robot and the patient. Several studies utilized optical tracking to relate preoperative images to the robot [7–10]. Nelson et al. [11] used a laser scanner to register a preoperative 3D US acquisition to the current position of the breast. The advantage of using just preoperative imaging is that the trajectory planning is not influenced or restricted by, e.g., US probe position. However, the procedure lacks real-time information to correct for deformations. Several studies utilized real-time US guidance as well. The position of the US probe with respect to the needle can be tracked optically, calculated based on joint sensors of the robot(s) holding the probe and/or the needle, or measured if the position of the US probe is static with respect to the needle base frame [7, 12–15].

Additionally, there are several approaches to needle insertion under US guidance. Liang et al. [14] presented a six-DOF robot holding a 3D US probe with the needle fixed to the probe. Mallapragada et al. [16, 17] presented a needle which had a fixed insertion orientation relative to the probe, but manipulated the tissue. Other studies presented setups in which the needle/needle guide has some degrees of freedom in the image plane of the US probe [13, 15, 18–21]. In some cases, the needle had DOFs out of the US plane as well or the US probe had degrees of freedom also [22–24]. If the needle moves independently of the US probe, there are more options for targeting lesions. However, if the needle moves out of the US plane, the US feedback is less accurate.

The above-mentioned studies show that the introduction of robotics to the biopsy workflow is advantageous for the accuracy of the procedure. However, to truly benefit from developments in the area of robotics such as the medically certified robotic arms, there is the need for an all in one solution. If one tool enables a robotic arm to autonomously perform all steps of the breast biopsy, the system becomes less complex and expensive, and inter-system calibration errors are ruled out. This will lead to a higher accuracy and faster acceptance in the medical world [25]. The aim of this paper is to present the design of an end-effector (EE) for utilization in a robot-assisted breast biopsy. The EE contains an actuated needle guide which directs the needle to a specified target within the US plane. The needle insertion is performed by the radiologist, which assures a human is still in control during the invasive step. The EE tracks the insertion and mechanically stops the needle at the specified depth. Utilizing the proposed system, MR-detected lesions may be targeted by a US-guided biopsy based on a registration step, which is less invasive than an MR-guided biopsy. Furthermore, biopsies can be consistently and reliably performed independently of the clinical background of the person performing the biopsy.

The paper is structured as follows: in “Design analysis” section, an analysis of the design constraints is presented. “End-effector” section presents the proposed and implemented design. “Experimental validation” section presents the measurements performed to characterize the system, and in “Discussion” section, the results are discussed. The paper concludes with “Conclusion and recommendations” section.

## Design analysis

**Fig. 1:**
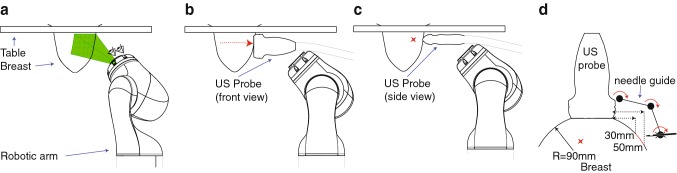
Robot-assisted biopsy workflow. **a** The robot scans the breast with cameras and registers the breast surface by projecting light or recognizing markers. **b** The robot scans the breast with a 2D US probe for 3D US volume reconstruction. **c** The robot visualizes the target in the US image. **d** The robot targets the lesion by aiming the needle guide to the correct location. In situations **b** and **c**, an angle of 45° of the probe relative to the flange is beneficial to navigate closely to the chest wall/patient table

The envisioned robot-assisted US-guided biopsy procedure consists of several phases (Fig. 1). First, a breast MRI is acquired in prone position. Then, the patient is positioned in prone position over the robot. This reduces motion artifacts and simplifies registration with the preoperative MRI scan. Multi-modality markers, visible in MRI, US and on camera, were attached to the breast.

The robot determines its position relative to the breast by moving around the breast and detecting the markers with cameras attached to the end-effector (Fig. 1a). The MRI data are then registered with the optical data. The markers’ relative positions and projections of a projector can be used for possible deformations compared to the preoperative MRI data.

Subsequently, the robot scans the breast surface with a 2D linear probe to acquire 3D US data. The volume is built up by streaming the 2D images with their corresponding position data to a reconstruction algorithm. It is important to navigate closely to the bed to optimize the scanning area. Therefore, the probe should be tilted with respect to the robot flange (see Fig. 1b).

The needle tip should be within the field of view (FOV) of the US transducer during insertion. This allows for real-time image feedback of both the needle tip and tissue deformations. The needle tip should be aligned with the lesion in the breast and approximately parallel with the transducer array of the US probe for needle visibility. Therefore, the needle will be inserted approximately 3–5 cm from the edge of the transducer. Furthermore, the needle is preferably inserted parallel to the chest wall because this reduces the risk for a pneumothorax. Due to these requirements, the anticipated pose of the probe during a biopsy is as shown in Fig. 1c.

If the US probe is correctly placed on the breast surface, the lesion will be a point in the 2D US image. The orientation and position of the needle guide are determined by the target and the insertion position. Therefore, a three degree of freedom (3DOF) articulated needle guide suffices to correctly aim the needle toward the lesion in the US image plane (Fig. 1d). The method to determine the joint angles on the basis of the needle guide’s position and orientation is described in [26]. The desired workspace of the manipulator is defined by the needle insertion rules and the diameter of the female breast, which is approximated to a maximum of 18 cm [27]. The needle guide should successfully target lesions with a size ranging from 4 to 10 mm. This includes lesions that are difficult to detect on US images but can be recognized on MRI [28].

The needle will be inserted in the breast through the needle guide, which limits the movement of the needle to the direction of insertion. The needle guide should stop and hold the needle at the desired depth, regardless of needle length and diameter. The brake should exert forces higher than the insertion forces to stop the needle. These forces will have a range of 0–3.5 N [29, 30]. Preferably, the mechanism is substituted or sterilized easily after usage.

## End-effector

### Design

**Fig. 2:**
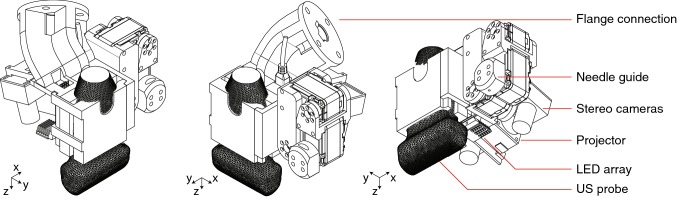
Isometric projections of the end-effector design. The US probe tip is rotated 45° w.r.t. the robot flange around both *x-* and *y*-axes. Further indicated are the needle guide, stereo cameras, projector, LED array and the US probe

An overview of the proposed end-effector design is shown in Fig. 2. The design was adapted for a KUKA MED 7 R800 (KUKA GmbH, Germany) and optimized for the phases described in the previous section.

The US probe is rotated relative to the robot flange—the tool mounting surface—to move close to the patient table in both the scanning and biopsy phase. Different probe types can be connected to the end-effector by exchanging the holder.

Cameras (KYT-U200-SNF01, Kayeton Technology Co., Ltd, China) and a projector (SK UO Smart Beam, Innoio, S. Korea) are installed to support in the localization phase. The stereo camera has wide angle lenses (focal length 2.8 mm) to cover a wide area regardless of the proximity to the breast surface. The cameras are synchronized for accurate stereo vision on a moving frame. Two LED arrays are placed next to the cameras to support in segmentation of the colored markers. During camera scanning, the cameras segment the colored markers applied to the patient’s skin or phantom. When both cameras image the same marker, the position of the marker centroid relative to the cameras is determined. After scanning, the marker centroids relative to the robot are known, and are registered with the marker centroids selected in the MRI scan (or CAD data of a phantom). This way, the lesion location known in MRI or phantom coordinates can be transformed to robot coordinates.

**Fig. 3:**
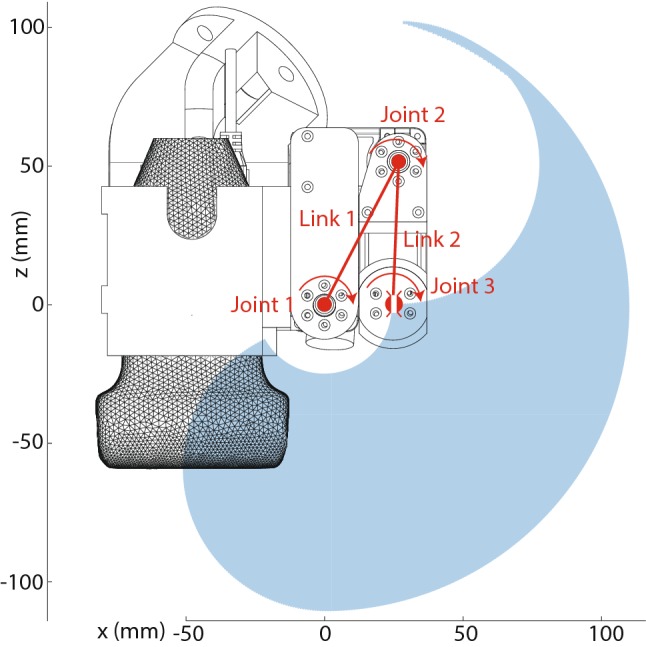
Three-DOF motorized needle guide. Link 1 is 57.09 mm and link 2 is 50.36 mm. The blue area indicates the workspace of the guide. The origin is located in the joint of the first motor

The needle placement is performed by a 3DOF manipulator consisting of two links and a needle guide. The motors have integrated controllers, have a range of 320° and a resolution of 0.325° (Herkulex DRS 0201, DST Robot Co., Ltd, S. Korea). Figure 3 highlights the 3DOF manipulator and its workspace. The maximum Euclidean error between the needle tip and the target in the range *x* = [− 25 25] mm and *z* = [− 15 − 45] mm is expected to range from 0.7 to 1.1 mm, based on the motor accuracy and the forward kinematics of system. The error increases as the distance between the needle guide and the lesion increases.

A printed circuit board (PCB) integrates a microcontroller (MCU) (ESP8266, Espressif Systems, China), supplies for the cameras, the picobeamer and the motor, LED drivers and communication with the robot controller. The MCU was programmed in the Arduino IDE (Arduino AG, Italy) to take serial commands from the robot controller and to control the motors, LEDs and the needle stop. The board has separate supplies for the microcontroller and the motors such that the robot controller can shut down the motors in case of emergency, while the communication with the end-effector continues.

**Fig. 4:**
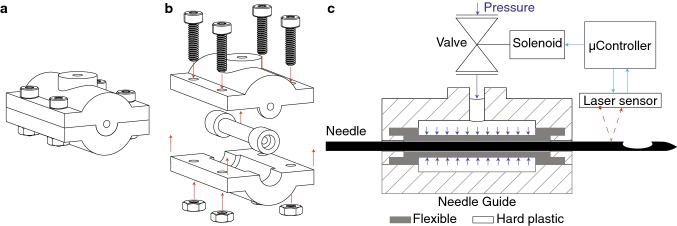
**a** The needle stop. **b** An exploded view of the needle stop. **c** A schematic diagram and a cross section of the needle stop. A laser sensor measures the needle position, and the microcontroller controls the pressure with a solenoid operated valve based on this position

An overview of the needle stopping system is shown in Fig. 4. The needle movement is limited to the direction of insertion by matching the guide diameter with the needle diameter. The guide was partly made of a hard plastic, which forms a chamber together with a more flexible plastic. The needle is stopped by pressurizing the chamber and deforming the flexible part of the guide. This creates friction forces which stop the needle. The following equation relates the change in inner radius *δr* (m) of a tube to the pressure difference on the inner and outer wall and its material properties [31, 32]:

1 $$ \delta r = \frac{1 - \nu }{E}\left( {\frac{{a^{2} p_{\text{i}} - b^{2} p_{\text{o}} }}{{b^{2} - a^{2} }}} \right)r + \frac{1 + \nu }{E}\left( {\frac{{a^{2} b^{2} \left( {p_{\text{i}} - p_{\text{o}} } \right)}}{{b^{2} - a^{2} }}} \right)\left( {\frac{1}{r}} \right), $$

in which *p*_o_ and *p*_i_ are the pressures on the outside and the inside of the tube (Pa), *r* is the initial radius of the tube (m), *E* is the Young’s modulus of the material (Pa), *ν* is the Poisson’s ratio of the material, and *a* and *b* are the inner and the outer radius of the tube (m). For a tube with an inner radius of 0.75 mm and pressures in the range of 0–6 × 10^5^ Pa, a wall thickness of 0.75 mm is sufficiently small to enable clamping the needle. A laser sensor (PAT9125, PixArt Imaging Inc., Taiwan) measures the needle displacement during insertion with a resolution of 20 μm. Based on the forward kinematics of the system, the MCU determines the position of the needle tip during insertion. The controller opens a pneumatic valve (PV3211-24VDC-1/8, FESTO Didactic GmbH & Co. KG, Germany) once the needle tip has reached the target.

### Realization

**Fig. 5:**
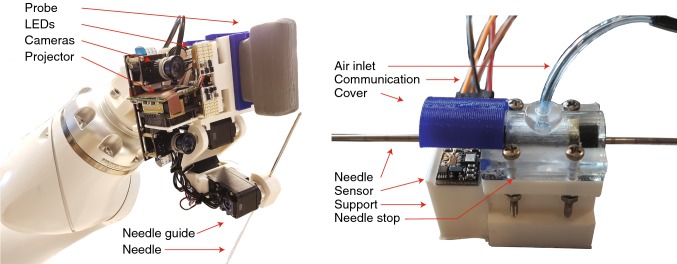
Left: the end-effector. Right: the needle stop. Red arrows indicate the relevant parts

Figure 5 presents the assembled EE. The left picture shows the EE with red arrows indicating the relevant parts. Similarly, the needle stop is shown on the right.

All structural parts, e.g., the links and the housing, of the end-effector are printed by fused deposition modeling printers—A Fortus 250MC (Stratasys Ltd., USA) and an Ultimaker S5 (Ultimaker, The Netherlands). The materials used are acrylonitrile butadiene styrene (ABS) (ABSplus, Stratasys, Ltd., USA) and polylactic acid (PLA) (Ultimaker, The Netherlands).

The needle guide is printed utilizing an Objet Eden 260VS (Stratasys Ltd., USA). The hard plastic is VeroClear (Stratasys Ltd., USA), whereas the flexible plastic is Agilus Black (Stratasys Ltd., USA).

## Experimental validation

### Experimental methods

**Fig. 6:**
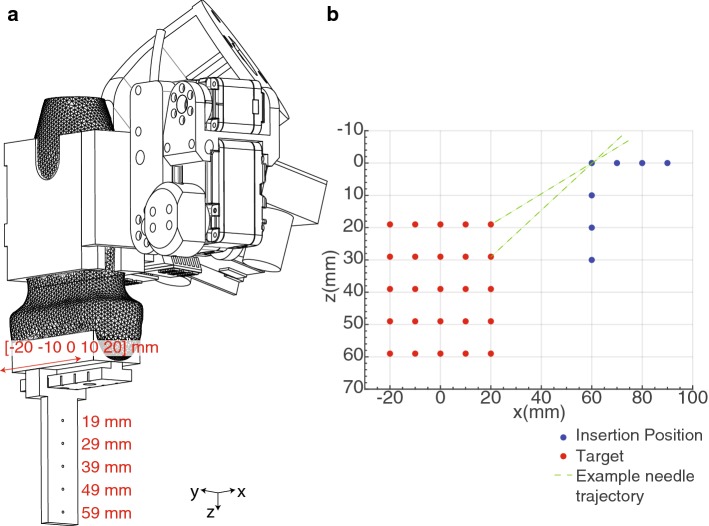
**a** Setup for measuring the accuracy and precision of the needle placement. **b** Set of targets and virtual insertion positions. The needle trajectory goes through one blue and one red point

An experiment was designed to verify the accuracy and precision with which the needle guide can guide the needle to a coordinate in the US image (Fig. 6). This experiment was performed in air to exclude the influence of tissue. The setup consisted of a mock-up US probe adapted to hold a displaceable plate with five targets indicating *z* = [19 29 39 49 59] mm. This plate was fixed on five marked locations, being *x* = [− 20 10 0 10 20] mm. This made a total of 25 targets (red dots, Fig. 6b). Each target was approached from seven insertion positions (blue dots, Fig. 6b). For every combination of target and insertion position, the needle was inserted, and the position on which the needle was in contact with the plate was recorded. A measurement accuracy of 0.5 mm was achieved utilizing a millimeter grid paper on the plate. Every combination of insertion and target position was performed five times. A needle with a conical tip (MRI IceRod™, Galil Medical Inc., USA) was used for optimal measurement accuracy. A MATLAB script (The MathWorks, Inc., USA) commanded the motor positions and saved the measured values.

The accuracy of the needle stop is defined by how well the needle is stopped at a specified depth. Therefore, the needle was inserted ten times for different depths, *d*_set_ = [30 50 70 90] mm. The depth, at which the needle was stopped, was measured using a micro-manipulator which was moved toward the tip of the needle until the sensor on the needle guide measures contact. The measurement accuracy was approximately 10 µm. Furthermore, the holding force was determined for pressures of [2 4 6] bar using a spring balance.

**Fig. 7:**
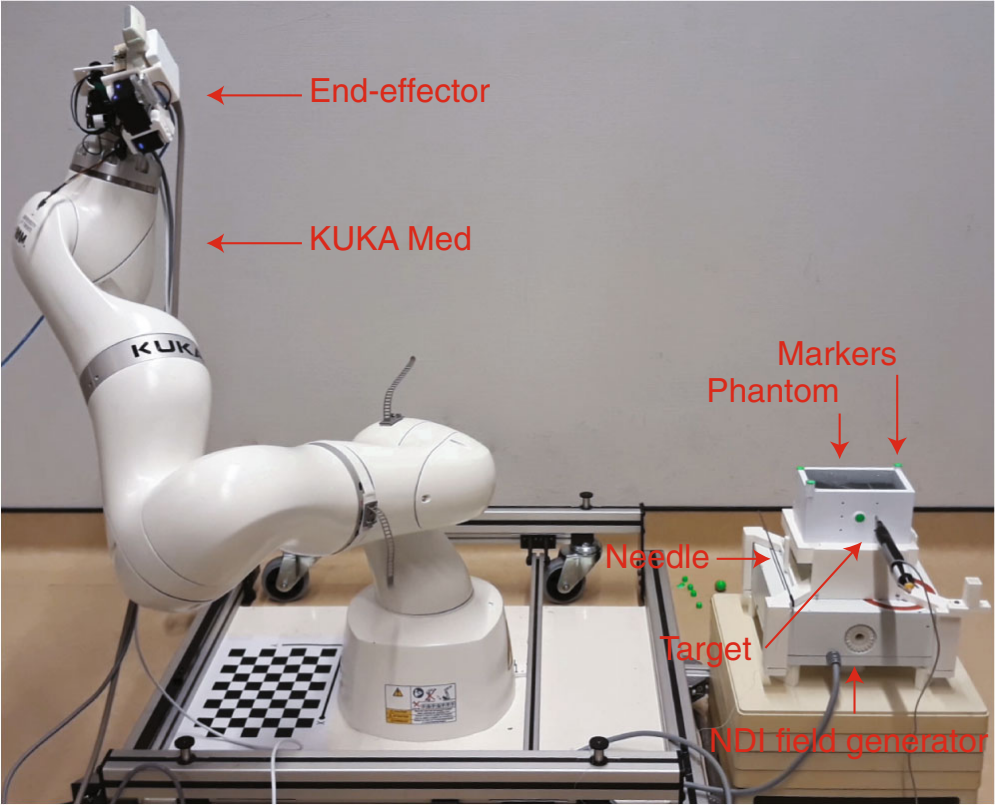
The experimental setup is comprised by a KUKA MED with the EE attached, a phantom with five markers placed over an NDI field generator, a target formed by an EM tracker and a needle with an integrated EM tracker

A third experiment was designed to determine the system accuracy (Fig. 7). The accuracy of the system is determined by how well the system targets a point specified in preoperative data. In a simplified setting, the CAD model of the phantom functions as preoperative data with a known shape, known marker positions and a known lesion position. For this, a cuboid phantom (6 × 6 × 11 cm^3^) was constructed from candle wax (CREARTEC trend-design-GmbH, Germany). The top of a grinding sponge was integrated in the bottom to avoid back-scattering of the US signal. The phantom was placed over and registered with an Aurora tracker (Northern Digital Inc., Canada). An electromagnetic (EM) tracker (Part nr: 610065, Northern Digital Inc., Canada) is placed inside the phantom to function as the lesion, and its location with respect to the phantom is precisely known. Now, the EE was connected to a KUKA MED 7 R800. A VF13-5 linear US probe (Siemens AG, Germany) was attached to the EE and connected to an X300 US system (Siemens AG, Germany). The robot retrieved the lesion position in robot coordinates by scanning the phantom with the cameras, determining the marker positions with respect to the robot and then registering the phantom with the robot-space. After registration, the robot moves to the phantom to perform the biopsy procedure. A custom biopsy needle was produced utilizing a metal tube with an outer diameter of 2 mm and an inner diameter of 1.6 mm and equipped with an EM tracker (Part nr: 610059). The needle is inserted to the specified position, and the Euclidean distance between the two sensors is recorded to determine the accuracy. The procedure is performed in supine position because the bed interferes with the signal of the Aurora system. The procedure was performed five times each for targets at a depth of 32.5 mm and 50 mm.

### Results

**Fig. 8:**
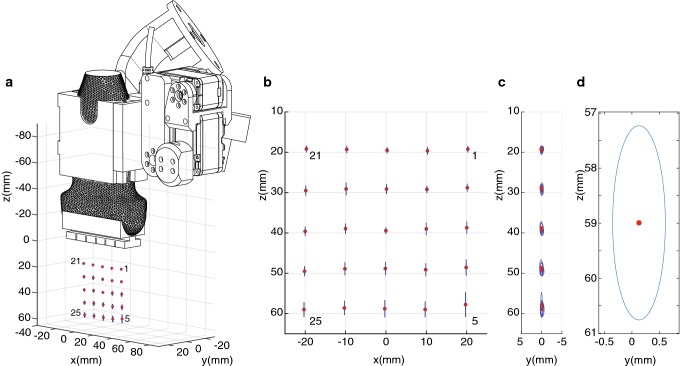
**a** The measured points plotted with the end-effector. **b**, **c** The measured points plotted in the *xz*- and the *yz*-planes, respectively. **d** The position which was targeted the least precise

The needle guidance experiment was performed five times, of which the first dataset was used to determine the linear transformation between the measurement results and the initially targeted positions. This transformation is applied to the rest of the data, and Fig. 8 shows the results. The red dots show the mean position for every target, while blue ellipses indicate the standard deviation in *z*- and *y*-directions. The mean error in *y*-direction and *z*-direction was 0.1 ± 0.36 mm and 0.3 ± 1.5 mm, respectively. Target 25 was targeted the least precise, with a standard deviation of 0.48 mm and 1.76 mm in *y*- and *z*-directions, respectively. Furthermore, target 5 had the largest standard deviation in *z*-direction, being 3.0 mm.

Table 1 presents the results of the needle clamp experiment. During a calibration step, the bias of the micro-manipulator relative to the needle guide (1.77 mm) was removed, and the resolution of the sensor was adjusted to 19.67 μm by means of a linear fit. The accuracy in the tested range was 0.100 mm (maximum error 0.89 mm). The holding force was determined to be 3.5–6 N.

**Table 1:** Top: the set and measured needle depths. Bottom: the applied pressure and the corresponding holding force

Set (mm)	30	50	70	90
Measured avg. (mm)	30.18	50.00	70.02	90.20
Min (mm)	29.75	49.82	69.89	90.05
Max (mm)	30.89	50.26	70.18	90.35

Pressure (bar)	2	4	6
Hold force (N)	3.5	5	6

**Fig. 9:**
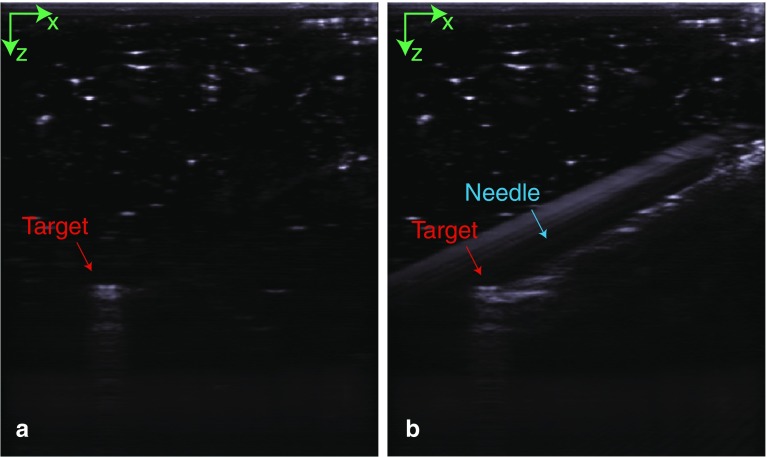
**a** The US plane containing the target. **b** The US plane containing the target after needle insertion

Table 2 presents the results of the phantom experiment. The Euclidean distance, *d*_Euc_, between the needle tip and the target is 3.21 mm on average. The normal distance, *d*_norm_, describes the shortest distance from the target to the needle trajectory and is 3.03 mm on average. The root-mean-square distance, *d*_marker_, between the marker centroids as segmented by the cameras and modeled phantom after transformation is 1.74 mm. Figure 9 shows how the metal tracker and the needle insertion were visible on the US image.

**Table 2:** Distance, *d*, the Euclidean distance, *d*_Euc_, and the normal distance, *d*_norm_ between the needle tip and the target, and the Euclidean distance between the markers after registration in the phantom experiment

	Needle					Marker
	*d* (*x y z*) (mm)			*d*_Euc_ (mm)	*d*_norm_ (mm)	*d*_Euc_ (mm)
Mean	1.03	− 2.62	− 0.11	3.21	3.03	1.74
Min	0.70	− 2.28	0.01	2.38	2.04	1.59
Max	2.49	− 3.70	− 1.57	4.72	4.61	1.85

## Discussion

An EE for a robotic arm was designed to perform a robot-assisted breast biopsy workflow: registration, 3D volume acquisition and the US-guided biopsy. The presented EE integrates all necessary features in a small package. The 45° angle of the US probe relative to the flange allows the robot to reach the breast near the chest wall during both the scanning and the biopsy phase. In a simplified setting, it was shown that pre- and intra-operational data can be registered utilizing the cameras and the LED arrays on the EE. Although not shown here, the picobeamer can help adding a deformable registration to the procedure. The 3DOF needle guide successfully assists the radiologist in targeting a lesion location defined preoperatively.

Both in-air and phantom experiments were performed to determine the needle placement accuracy. The in-air experiments showed that the needle is accurately guided to a predefined position in the US plane, and the needle is accurately stopped at a predefined depth. The phantom experiment showed that the needle trajectory has a mean normal distance of 3.03 mm to the target. It is shown in Table 2 that a large contribution to this error is in the *y*-direction, out of the US plane, while the in-plane errors are similar to the in-air experiments, which were focused on needle guidance and stopping accuracy. Furthermore, Table 2 shows that the camera segmentation has an error in the millimeter range. As a certain force was needed to insert the target in the phantom, it is suspected that this caused a small error in the phantom to field generator registration. Other factors influencing the error metric could include the accuracy of the calibrations of the needle guide, the US probe and the cameras with respect to the robot flange and the inter-camera position. All in all, the EE has a similar accuracy as the cited studies (0.25–3.44 mm [10, 22]), and for the system, it is feasible to target lesions in the range of 4–10 mm in the future.

Considering Fig. 7, the standard deviations are relatively large compared to the mean errors since the motors have backlash in the gears. Additionally, the printed parts do not provide the same rigidity as, e.g., metal parts. Furthermore, target 5 has a relatively large standard deviation in the *z*-direction because the needle reaches this target under a sharp angle. Small deviations in target placement and the insertion angle cause a relatively large variation in Euclidean distance errors. Target 25 is targeted the least precise since this target is located the farthest away from the needle guide. Both positions will not be used in real-life scenarios; as for optimal needle and target visibility, the target is normally located more toward the center of the US image.

The system has several advantages: due to the markers recognition, the biopsy site can be marked on preoperative images and the correct biopsy site is found. Due to the needle guide, the radiologist remains in control of the insertion yet has a robotic biopsy accuracy. The physician has valuable feedback when puncturing the skin and other tissue boundaries due to the frictionless movement of the needle. The displacement sensor’s accuracy is satisfactory, considering that in the range of 30–90 mm, the stopping system has an accuracy of 0.100 mm. The laser is located away from the needle, so the needle guide is easily replaced after performing a biopsy or when changing the needle diameter. Furthermore, the system works independently of the needle length. Also, when power is lost, the needle is released, and in case of emergency, the practitioner can remove the needle by overcoming the clamping forces. This makes the system safe to use in a clinical environment.

In the current setup, possible deformations were not considered but this was not necessary since the target position was static. In future experiments in which the lesion can be displaced by the needle insertion, this should be implemented. This may be done utilizing simulations or by tracking the needle and deformations in the US image. Needle tracking may also decrease the influence of backlash and the rigidity of the system by providing feedback. Further improvements include changing the material of the clamping mechanism of the needle stop, which is too brittle. Due to the brittleness, it is difficult to make the mechanism airtight and durable. However, this did not influence the working principle of the needle stop.

For clinical application it is important that the procedure is sterile. During camera scanning, the EE is not in contact with the patient. During needle insertion, the needle guide is in contact with the needle, and thus this part will be a disposable. During the procedure, a US transparent sheet can cover the setup to create a sterile environment.

## Conclusion and recommendations

This paper introduced an EE for a robotic manipulator to assist the radiologist in acquiring US breast scans and performing the US-guided biopsy. The 3DOF needle guide with needle stop gives radiologist robotic accuracy yet the radiologist is in control since needle insertion is not robotized.

The accuracy and precision of the 3DOF needle guide were determined experimentally both in-air and on a phantom. The results look promising and indicate that targeting lesions in the size range of 4–10 mm is feasible.

The results of this study are an example of how to integrate different aspects of robotic US scanning and robot-assisted biopsy in one functional device.

The following improvements are recommended to further increase the accuracy and precision: implementing standardized sequences for the inter-camera, the camera to flange, the US probe to flange and the needle guide to flange calibration. Installing backlash-less motors like harmonic drives to increase precision and stability of the needle guide. Changing the 3D printed plastics for more rigid CNC machined parts which will ensure the rigidity of the system and stability of calibration parameters over time.

## Electronic supplementary material

Below is the link to the electronic supplementary material.

Supplementary material 1 (STL 743 kb)

Supplementary material 2 (STL 525 kb)

Supplementary material 3 (STL 2013 kb)

Supplementary material 4 (STL 764 kb)

Supplementary material 5 (STL 101 kb)

Supplementary material 6 (STL 164 kb)

Supplementary material 7 (STL 322 kb)

Supplementary material 8 (STL 101 kb)

Supplementary material 9 (DRL 0 kb)

Supplementary material 10 (GBR 9 kb)

Supplementary material 11 (GBR 0 kb)

Supplementary material 12 (GBR 1 kb)

Supplementary material 13 (GBR 0 kb)

Supplementary material 14 (GBR 17 kb)

Supplementary material 15 (GBR 0 kb)

Supplementary material 16 (GBR 3 kb)

Supplementary material 17 (STL 1117 kb)

Supplementary material 18 (STL 432 kb)

Supplementary material 19 (STL 523 kb)

Supplementary material 20 (MP4 129633 kb)
